# Dissemination of a CBPR Study With Alaska Native Elder Advisory Committee members

**DOI:** 10.1093/geroni/igaf122.1287

**Published:** 2025-12-31

**Authors:** Lena Thompson, Steffi Kim, Marcelo Quinto, Jordan Lewis

**Affiliations:** University of Alaska Fairbanks, Fairbanks, Alaska, United States; University of Alaska Anchorage, Anchorage, Alaska, United States; Alaska Native Brotherhood, Juneau, Alaska, United States; Center on Aging, Thompson School of Social Work & Public Health, University of Hawai’i at Manoa, Honolulu, Hawaii, United States

## Abstract

Historically, unethical research practices with American Indian and Alaska Native communities has left community members feeling distrustful of Western research institutions. While the scientific community has made improvements in fostering community participation in the designing and analyzing of research, community participants are often left out of dissemination efforts. Dr. Lewis and his research team have worked to build healthy relationships with Alaska Native communities through an 18-year Community-based participatory research study exploring successful aging among Alaska Native communities. To disseminate study findings, the research team worked with two Alaska Native Elder Advisory Committees (EAC) in Southeast and Interior Alaska to conceptualize, plan, and carry out dissemination efforts locally, nationally, and internationally. In addition to peer-reviewed manuscripts and conference presentations, the study team worked with Elders to plan community presentations and discussions, short videos, and products that could be worn and used in the community, such as sweatshirts and berry buckets with the study logo. Elders presented knowledge gained though the study at an international Arctic conference. Opportunities like this include community members beyond local dissemination efforts to receive international recognition of their community and scholarly efforts. EAC members described feeling grateful for opportunities to travel and were excited to disseminate through videos and products to engage youth. Community participation in dissemination efforts is a key aspect to conducting responsible Community-based participation research. Dissemination efforts should find balance between meeting community needs and grant/academic requirements. Creative dissemination efforts, such as products and videos can help findings reach a wider audience.

